# How Many Protein-Protein Interactions Types Exist in Nature?

**DOI:** 10.1371/journal.pone.0038913

**Published:** 2012-06-13

**Authors:** Leonardo Garma, Srayanta Mukherjee, Pralay Mitra, Yang Zhang

**Affiliations:** 1 Department of Computational Medicine and Bioinformatics, University of Michigan, Ann Arbor, Michigan, United States of America; 2 Biocenter Oulu and Department of Biochemistry, University of Oulu, Oulu, Finland; University of South Florida College of Medicine, United States of America

## Abstract

“Protein quaternary structure universe” refers to the ensemble of all protein-protein complexes across all organisms in nature. The number of quaternary folds thus corresponds to the number of ways proteins physically interact with other proteins. This study focuses on answering two basic questions: Whether the number of protein-protein interactions is limited and, if yes, how many different quaternary folds exist in nature. By all-to-all sequence and structure comparisons, we grouped the protein complexes in the protein data bank (PDB) into 3,629 families and 1,761 folds. A statistical model was introduced to obtain the quantitative relation between the numbers of quaternary families and quaternary folds in nature. The total number of possible protein-protein interactions was estimated around 4,000, which indicates that the current protein repository contains only 42% of quaternary folds in nature and a full coverage needs approximately a quarter century of experimental effort. The results have important implications to the protein complex structural modeling and the structure genomics of protein-protein interactions.

## Introduction

The protein universe refers to a collection of all proteins across all organisms in nature [1]. In 1992, there were only 887 protein structures in the Protein Data Bank (PDB) which could be categorized into 120 different tertiary folds. Chothia [2] noticed that about 1/4 of the entries at the EMBL/SwissProt sequence databank were homologous to the 120 folds, and 1/3 of the genome sequences presented in the sequence databank. He thereby suggested that the number of protein tertiary folds in the protein universe should be limited and around 1500 (∼120×3×4). Amazingly, this simple estimation stood well the test of time and lies at the center of the subsequent estimation range (1000–2000) using more elaborate methods based on much larger datasets [3], [4], [5], [6]. At present, the PDB has over 70 k structures, which has been argued to be structurally complete [1], [7], [8], [9]. The structure set has been categorized into 1,195 folds by SCOP [10] in the 2009 release, consistent with the Chothia’s original estimation.

In contrast to the extensive studies of protein tertiary structural space, the quaternary structure space of protein-protein interactions is relatively unexplored. For example, the questions on whether the number of unique protein-protein complex structures is constrained and if yes, how many they are, have remained largely unanswered. Since most proteins perform their physiological functions via interaction with other protein molecules, the answers to these questions have practical applications in the understanding of protein-protein interaction specificity and protein-protein networks [11]. Meanwhile, the template-based methods have recently demonstrated promising power in protein complex structural modeling [12], [13], [14]; the completeness of the quaternary structure space is of important implications to the studies of protein-protein docking and structure prediction [15], and the forthcoming structural genomics of protein-protein interactions [16].

Exploration of the quaternary structure space has been mainly hampered by the relative dearth of protein-protein complex structures in the PDB library, and the lack of an unambiguous definition of protein quaternary structural folds and efficient methods to compare and categorize protein-protein complex structures. Among limited attempts, Aloy and Russell [17] exploited the protein-protein interaction data from high-throughput genomic data to estimate, based on the assumption that homologous proteins (with a sequence identity >25%) should participate in similar interactions, that the number of unique protein-protein interactions is around 10,000. Although the estimation could be meaningful for the complex homologous families, it is often observed that proteins of different sequences (not belonging to the same homologous family) have similar complex structure and interface interactions. Thus, the Aloy-Russell calculation may overestimate the protein-protein interaction space if the protein-protein interactions are counted at the structural level.

Here, we present a systemic study of a representative set of protein-protein complex structures in the PDB, with all structural pairs compared by a recently developed protein complex structural alignment algorithm, MM-align [18]. The complex structure similarity is evaluated by a newly defined reciprocal TM-score, rTM-score, which is sensitive to both the monomeric structure similarity of the individual subunits as well as the relative chain orientation of the complexes. The number of protein-protein structural families (called ‘quaternary fold’ throughout the paper) in nature is then estimated from the sequence families and structural folds currently present in PDB, under the assumption that the current PDB is a random subset of the structural universe. Since dimeric protein-protein interaction is the basic unit of all higher-order oligomers, our calculation is focused on the dimer structures.

## Methods

### Structure Dataset Preparation

A non-redundant dimeric structure library was screened from DOCKGROUND [19] with a pair-wise sequence identity ≤90%, after an initial filtering to remove irregular structures and complexes with alternate binding modes. Since this work focuses on protein-protein dimers only, we split higher-order complexes into dimers by taking all possible dimeric combinations of protein chains in the complex.

For the counting of physically (and biologically) meaningful protein-protein interactions, it is important to focus only on bona fide dimers in our dataset. For this purpose, DOCKGROUND has screened its complexes from the PDB Biological Unit files in order to ensure that crystallization artifacts are removed. Second, we eliminated all complexes with <30 interface residues and/or <250 Å^2^ buried surface area. These procedures result in a total set of 7,616 non-redundant dimeric protein structures for our consideration (as of December 2011).

We also attempted to apply the computational methods, including IPAC [20], DiMoVo [21] and NOXclass [22], to predict whether the crystal contacts are energetically stable enough for standalone interactions. Although the prediction results vary among different methods, we found that 2,692 (∼74%) out of the 3,629 representative complexes from each of the quaternary families (defined later) were deemed as bona fide dimmers by all three methods. The remaining 937 structures are nevertheless all belonging to existing larger family clusters. In other words, the excluding of the 937 putative structures would not change the number of quaternary families but the size of some families. Here, to avoid the theoretical uncertainties in the dimer predictions, we will stick our calculations mainly on the 7,616 non-redundant complexes which were selected by the first two experimental filters.

### Protein Complex Structural Alignment Method

The pair-wise alignment of protein-protein complex structures is constructed by MM-align [18]. For two protein complexes (AB and A’B’), it searches for optimal alignments of both AB to A’B’ and AB to B’A’ and chooses the alignment with the highest rTM-score. At the first step, MM-align joins the C-terminus of the first protein chain with the N-terminus of the second chain and treats the combined “artificial monomer” as rigid-body alignment units.

At the second step, a set of five initial alignments are constructed, including (1) an alignment of secondary structure (SS) elements; (2) gapless threading of two complex sequences; (3) an alignment based on the sum of the SS score and the distance score matrix from the second initial alignment; (4) a gapless threading of the longest continuous segments in the complexes; (5) a scan of superimpositions of five-residue fragment pairs.

At the third step, a residue-residue distance similarity matrix *S_ij_*=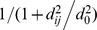 is derived based on the TM-score structure superposition of the initial alignments where *d_ij_* is the distance of *i*th residue in the first complex and *j*th residue in the second. A modified Needleman-Wunsch dynamic programming [23] is then implemented to identify the best alignments using the scoring matrix *S_ij_*. Based on the new alignment, a new scoring matrix is derived, on which a fresh alignment is generated again by dynamic programming. This procedure is repeated till a converged alignment is reached. Finally, the alignment with the highest rTM-score is returned.

### Assessment of Complex Structure Similarity

The similarity of protein tertiary structures is often evaluated by TM-score [24], which can be simply extended to the comparison of complex structures:
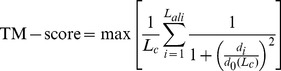
(1)where *L*_c_ is the total length of all chains in the target complex and *L*_ali_ is the number of the aligned residue pairs in the two complexes. *d_i_* is the distance of *i*th pair of Cα atoms after the superposition. 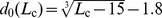 is a length-dependent scale to normalize the distance so that the TM-score of random complex structures is independent of the protein size. max[…] indicates the optimal superposition to maximize the TM-score value.

For complexes, TM-score in Eq. 1 can be factorized as two additive parts from two chains:
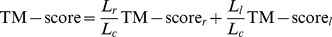
(2)where *L_r_* and *L_l_* are lengths of the receptor and ligand, respectively; TM-score_r_ and TM-score_l_ are their TM-scores calculated based on the same rotation matrix of the complex superposition. Therefore, one flaw of TM-score, when used to compare complex structures, is that it becomes more sensitive to the tertiary structure of the monomers, due to the linear dependence of the monomer TM-scores. For example, for a pair of homodimers, if the structure of one chain is identical, the TM-score is at least 0.5 even if the orientation of the other chain is completely different (see e.g. Figure S2a). For heterodimeric complexes, if one chain is much bigger than the other, the TM-score can be dominated by the structural similarity of the bigger chains regardless of the structure and orientation of the smaller chains because the weighting factor for the small chain (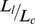) is too small in Eq. 2 (see e.g. Figure S2b). To overcome this drawback, we define a new score called reciprocal TM-score, or rTM-score, given by
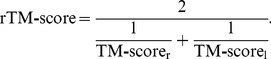
(3)This definition of rTM-score makes the score more sensitive to the overall structure similarity of the complex, i.e. the relative orientation of the component chains, rather than the individual monomer structures. For instance, if the structure or orientation of one chain is very different (i.e. TM-score_l_∼0), the rTM-score of the complex structure will be close to 0 even if the structure of another chain is identical (TM-score_r_∼1). In other words, two complexes have a high rTM-score only when both the monomer tertiary structure and the relative orientation are similar.

Quantitatively, for tertiary protein structures, it has been shown [25] that the posterior probability of TM-score of random protein structure pairs has a rapid phase transition at TM-score=0.5 and the structures of TM-score >0.5 approximately corresponds to the same protein folds as defined by SCOP [10] and CATH [26] databases. Similarly, we define rTM-score >0.5 as the complexes of the same interactions. Mathematically, this corresponds to two complexes which have two chains with the similar relative orientation and the similar folds (i.e. TM-score_r,l_ >0.5) according to Eq. 3.

In Text S1, we gave a more quantitative discussion on the relationship and difference between TM-score and rTM-score for protein complex structures (see Figure S1).

### Complex Structure Clustering

MM-align was used to compare each protein complex in the non-redundant complex library to all other complex structures. It returned an rTM-score as the measure of the structural similarity of the complex pairs. SPICKER [27] was then used to identify independent structure folds based on the rTM-score score matrix. First, a cluster center of the complex structures is identified which has the maximum number of structural neighbors, where a neighbor is defined if two complexes have an rTM-score >0.5. The first cluster (represented by the cluster center) and all the neighbors were removed from the library. The second cluster center was then identified which has the maximum number of neighbors in the remaining complex structures. The structures of the second cluster were removed again for identifying the third cluster. The process was repeated till only “orphan” complexes remained. The whole set of clusters consist of all the clusters of multiple members and the orphan complexes.

### Statistical Model for Estimation of the Universe of Complex Folds

Protein complexes solved in the PDB library is only a small subset of the complex universe in nature. Let’s suppose the numbers of complex folds in nature and in the PDB are *N* and *n*, respectively. At the sequence level, protein complexes can be categorized into homologous families and we suppose the numbers of complex families in nature and in PDB are *M* and *m*, respectively. Apparently, a fold can contain multiple families since it is well-known that different sequentially homologous families can have the similar structural folds.

If we suppose that the PDB structures are a random subset of nature, an assumption taken by many tertiary fold estimation models [2], [3], [5], [6], the probability of a family to be included in the PDB is λ=*m/M*. Thus, the probability of a fold having *Q* families in nature to be included in the PDB as a fold with *q* families is
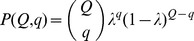
(4)Therefore, the expected number of the quaternary folds containing *q* quaternary families in the PDB can be calculated using the equation
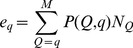
(5)where N_Q_ is the total number of quaternary folds containing Q quaternary families in nature.

Following the idea of the moment method of estimation [5], [28], we group the quaternary folds in nature according to their size, that is, the number of quaternary families they contain. Suppose that the group *G_I_* contains *X_I_* quaternary folds with sizes *K_I_* to *L_I_* (*L_I_* >*K_I_*) with each quaternary fold appearing with equal probability of 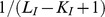, *I*=1,2, … *I*_max_. The observed quaternary folds in the PDB are grouped in a similar way, i.e. the group *g_i_* will include *x_i_* quaternary folds that comprise *k_i_* to *l_i_* quaternary families. Thus, the expected number of observed quaternary folds in the PDB in the group *g_i_* can be written as
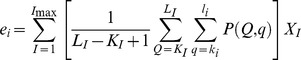
(6)If the expected value *e_i_* is replaced with the observed numbers in the PDB library, we get a linear equation for each of the *g_i_* groups that allow us to calculate *X_I_*, the number of quaternary folds of each group in nature. The total number of quaternary folds in nature, is then



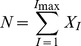
(7)To determine the values of *K_I_* and *L_I_*, which are needed for solving Eq. 6, we use the maximum probability principle method. Since quaternary folds consisting of more than 5 families represent a rare fraction (<5%) in the library, we divide the ensemble of quaternary folds in the PDB into 9 groups with *x_i_*>*x_i_*+1. The groups in nature were deduced according to the maximum probability principle, i.e. an observed quaternary fold with *q* quaternary families in the PDB should come from the quaternary fold with *T_q_* families in nature so that the probability *P*(*T_q_, q*) is maximal. Thus, the intervals for the values of *K_I_* and *L_I_* of the Groups 5 to 9 in nature can be set using following rules:



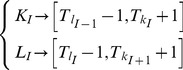
(8)where *k_I_* and *l_I_* are the boundaries of the corresponding groups in the PDB database. We note here that the indexes of groups are the same in the PDB and in nature. For the Groups 1 to 4, we have
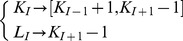
(9)Then we explored all the combinations of *K_I_* and *L_I_* according to Eqs. 8–9 for each group in nature and selected the ones that fulfilled the condition
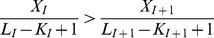
(10)for all values of I. From these, the set that provides the highest value for XI was chosen.

Once the number of quaternary folds *X_I_* in each group is calculated, the total number of estimated quaternary folds in nature can be easily calculated by Eq. 7. From these values, the predicted total number of quaternary families is then
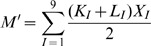
(11)


## Results

### Number of Observed Quaternary Families in PDB

Pfam is a standard database for protein domain families where each family is represented by a multiple sequence alignment (MSA) as searched by Hidden Markov Model (HMM) [29]. To identify the evolutionary families in protein-protein complexes, we followed a similar idea of iPfam [30], i.e. two complexes are classified into the same quaternary family if both subunits of the complexes belong to the same Pfam family. For example, complexes A-B and A’-B’ are in the same quaternary family if Chains A’ and A are in the same Pfam family and Chains B’ and B are also in the same family. For subunits which have multiple domains, the family of the largest interacting domain represented that of the whole chain.

Following this procedure, 7,523 out of the 7,616 complexes could be assigned to a Pfam family with an E-value <0.001, which results in 3,536 distinct quaternary Pfam families. The remaining 93 complexes did not return any Pfam hits for either subunit. These complexes shared a sequence identity <20% to each other and to all other complexes in the library, and hence were considered orphan families. Thus, 3,629 homologous families are obtained in total from the 7,616 protein-protein complexes based on evolutionary and sequence comparisons.

### Number of Observed Quaternary Folds in PDB

The structural types of protein-protein interactions is specified by rTM-score, a scoring function designed to simultaneously assess the similarity between the individual subunits as well as their relative orientation of two complexes (see Eq. 3). Given a pair of complexes, rTM-score was calculated by an extended version of MM-align [18] which is a complex structural alignment algorithm designed to identify the best structural match with the highest rTM-score (see METHODS).

**Figure 1:**
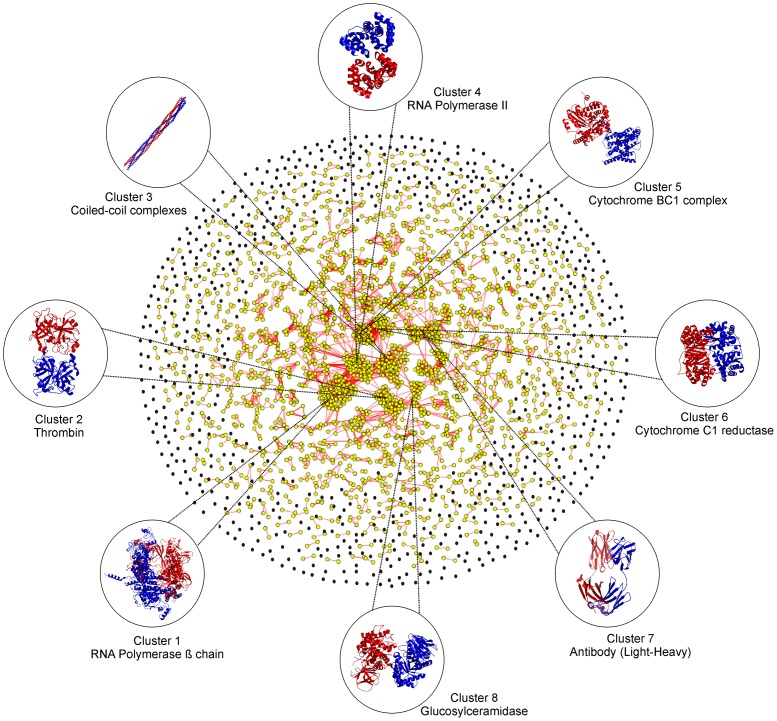
Graphical representation of all non-redundant protein-protein complex structures in the PDB. Each node represents a known complex structure and two nodes are connected by an edge if the rTM-score between the two structures is >0.5. The orphan nodes are shown in black while nodes which are connected by at least one edge shown in yellow. Representative examples from eight largest clusters are listed together with the protein name.

Using an rTM-score cutoff 0.5, the 3,629 quaternary families could be clustered by SPICKER [27] into 1,761 structural clusters, with the largest cluster containing 47 complexes (Figure 1). Remarkably, 60.6% of the clusters are single complex clusters or “orphans”, i.e. no other structures exist in the PDB library with an rTM-score >0.5 to any of these proteins. In contrast, if the tertiary structure fold is considered, almost all single-domain proteins in the PDB can have other non-homologous counterparts which share a similar fold [8], [31]. These data indicate that the current structure library is far from complete in the quaternary fold space.

Proteins of similar structure usually have similar function. Not surprisingly, it is observed that the proteins in each of our clusters present considerable convergence of function, although the sequence identity of protein complexes in one cluster can be as low as 9.9%. For instance, the biggest cluster consists of 47 members with the average and the lowest pair-wise sequence identity 41.6% and 17.7%, respectively. Despite the low sequence identity, all 47 structures are part of the RNA polymerase superfamily from different species. The second biggest cluster contains 34 complexes with the average and the lowest sequence identity 34.8% and 21.3%, respectively; all 34 are enzyme-inhibitor complexes where the enzyme is trypsin, thrombin or their derivatives (chymotrypsin, thrombinogen etc.). These complexes contain also the same GO term for “Tissue factor”. The third largest cluster consists of 32 coiled-coil complexes of the “mainly alpha” class of proteins. A graphical representation of all complex structures in our dataset has been shown in Figure 1 using Cytoscape [32]. Here, each complex structure in our dataset is represented by a node which are connected by an edge if the rTM-score between two nodes is >0.5.

**Figure 2:**
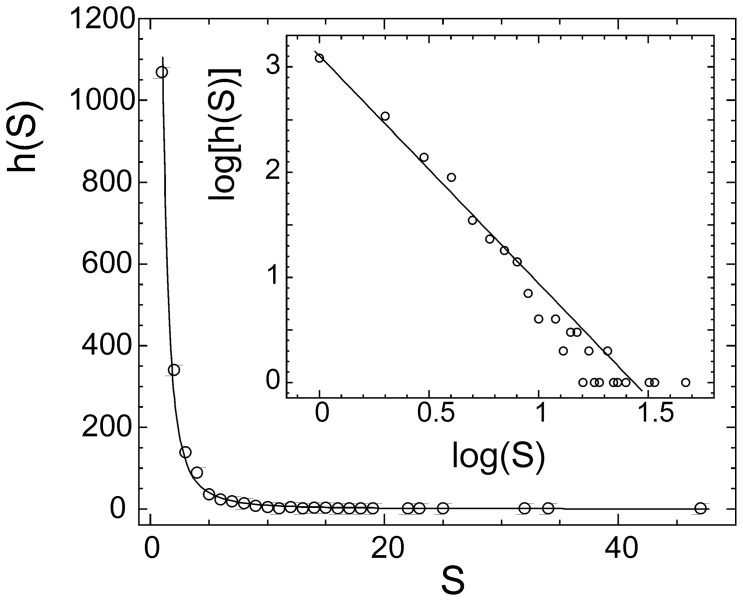
Histogram of complex structural clusters versus size of the clusters. The solid curve is the fitting result from Eq. 12. Inset: the same data drawn in logarithm scale.



Another intriguing trend that was observed is that the cluster size distribution follows a power-law dependence as shown in Figure 2. The best fit to the data results in

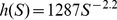
(12)where *h*(*S*) is the histogram of the structure clusters which have *S* complex members.

A similar power-law distribution has been extensively observed in the clustering of tertiary structures of protein domains [33], [34], [35], which was successfully explained by the cascade gene-duplication model [34], [35]. Since protein-protein complexes comprise of monomeric protein domains and the generation of protein complexes is closely interplayed with the evolution of individual protein molecules, the data shown in Fig. 2 and Eq. 12 may implicate a similar evolutionary mechanism involved in the protein-protein interaction generations.

### Estimation of Quaternary Folds in Nature

We use a statistical model (as outlined in METHODS) to estimate the number of quaternary folds, which assumes that the current PDB library is a random subset of the complex universe. First, to calculate the probability of a quaternary family to be included in the PDB, we need an estimation of the number of complex homologous families in nature (*M*). Using the SwissProt database with a sequence identity cutoff of 30%, Orengo *et al*. estimated the number of protein tertiary families as 23,100 [36]. This estimation is roughly consistent with the Pfam statistics as the number of Pfam families is 12,273 in current databases [29]; the number of Pfam families keeps growing and it was estimated that 38 k Pfam families are needed to cover the majority of UniProt sequences [37].

Bearing in mind that most dimeric complexes are composed of monomers that belongs to same tertiary families, it may be reasonable to assume that the number of quaternary families is similar to that of tertiary families. In fact, among the 3,629 dimeric families in the PDB, 2,106 (58%) are homodimers; the number of quaternary families in these proteins is identical to that of tertiary families. For heterodimers, if all component chains were non-homologous to each other, the number of quaternary families should have been half of that of the involved tertiary structural families. Actually, in the 1,523 heterodimer families in PDB, around 11% consist of homologous monomers with a sequence identity >70% and around 50% have monomers with sequence identity >30%. Thus, the number of quaternary families in heterodimers should be considerably higher than half of the tertiary families they contain. There are also some proteins which may not participate in any interactions but the number of these proteins should be neglectable since most proteins perform functions through interactions with other proteins [38], [39]. Therefore, the total number of tertiary families should be a reasonable approximation for that of the quaternary families.

**Figure 3:**
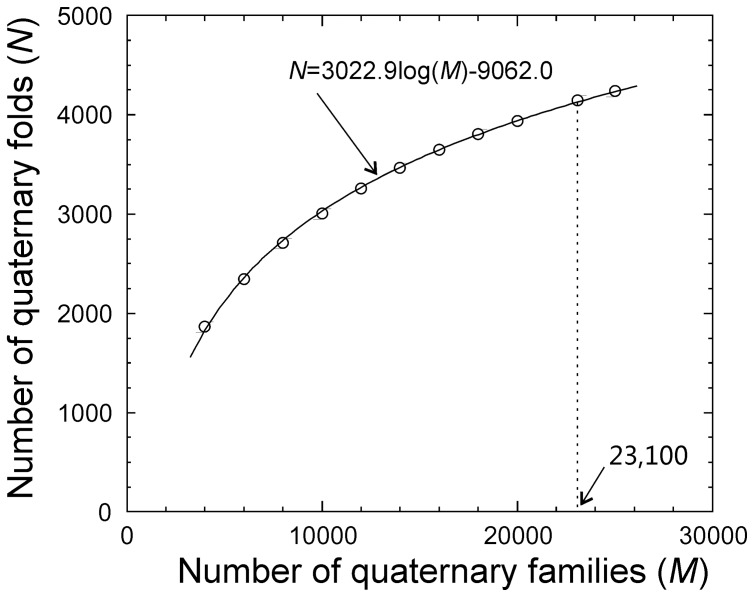
The estimated number of quaternary folds versus the number of quaternary families in nature. The solid curve is the fitting from Eq. 13 and dotted line indicates the number of quaternary families following Orengo *et al*. estimation.



In Table 1, we calculated the number of quaternary folds (*N*) using Eqs. 7–10 for a range of *M* values from 4,000 to 25,000. As observed in Figure 3, the values of *N* and *M* show a clear logarithmic dependence:

**Table 1:** Number of estimated complex folds for a range of numbers of complex families.

Number of Quaternary Families (*M*)	Number of Quaternary Folds (*N*)	Number of Predicted Quaternary Families (*M’*)
4,000	1,869	4,045
6,000	2,344	6,034
8,000	2,712	8,079
10,000	3,009	10,085
12,000	3,256	12,156
14,000	3,465	14,049
16,000	3,647	16,088
18,000	3,808	18,074
20,000	3,940	20,083
23,100	4,149	23,230
25,000	4,242	25,158

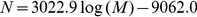
(13)If we take the number of monomer families by Orengo *et al*. and suppose the number of quaternary sequence families is the same as that of monomers, the probability for a quaternary family to be included in the PDB is λ=0.16 (=3,629/23,100) and the number of quaternary folds in nature should be 4,149. If taking the maximum Pfam number as the number of quaternary families (38,000), the number of expected quaternary folds is 4,782.

Since the number of the quaternary families can also be derived independently for any given *N*, as an examination of self-consistence of our calculations, we estimated the number of quaternary families (*M’*) assuming that the range of *N* predicted in the previous step are actual values existing in nature. In Column 3 of Table 1, we list the estimated values of *M’* calculated by Eq. 13, which is in close agreement with the arbitrary values of *M,* with a Pearson correlation coefficient=0.999.

To cross-validate our results as well as to verify the consistency of our definition of quaternary family, instead of using the Pfam family definition, the calculations were repeated by defining quaternary families based on pair-wise sequence comparisons, i.e. two complexes are in the same family if both chains have a sequence identity >30%. The cutoff of 30% in sequence identity has been extensively used as the homologous family cutoff for monomer proteins [40], [41] since the evolutionary conservation rate has a clear translation around 30% [42]. By using this definition, the 7,616 non-redundant protein complexes in the PDB could be classified into 3,793 sequence families which were further clustered by MM-align into 1,520 quaternary structural folds. A similar number of quaternary folds (4,302) was obtained if we suppose the number of quaternary families is similar to the number of tertiary ones in nature.

### When Protein-protein Complex Structure Library can be Complete?

**Figure 4:**
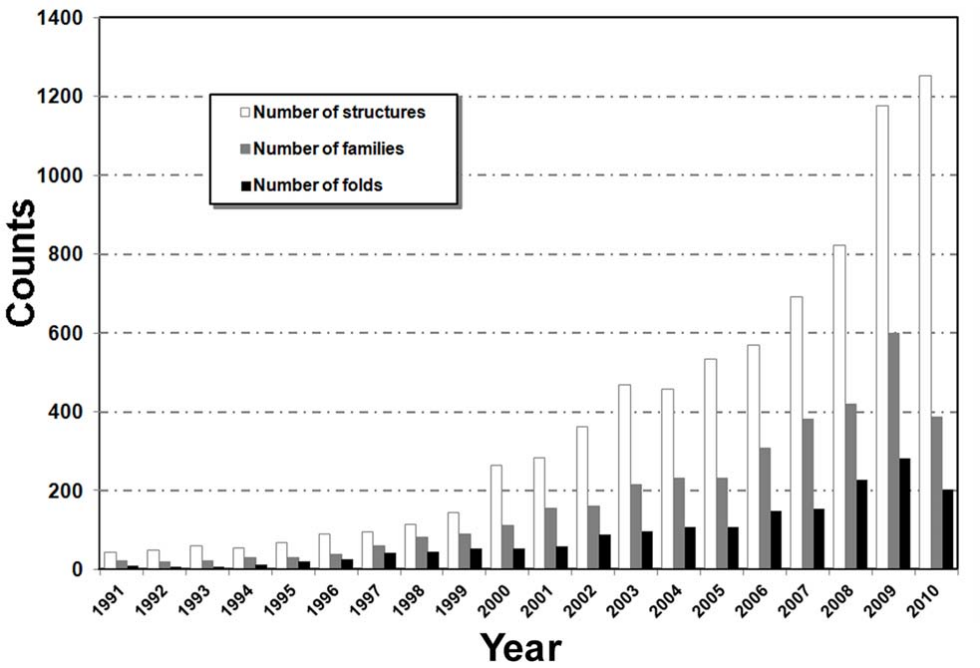
The number of new complex structure entries deposited per year in the PDB. Data are presented in terms of unique structures (sequence identity <90%), families (mapped with unique Pfam families), and folds (rTM-score <0.5).

The above analysis showed that the current PDB library accounts for <50% of total folds in nature. While the number of tertiary folds in PDB is approaching its completeness [1], [7], [8], [9], an intriguing question is when the majority of distinct protein-protein complexes can be solved. In Figure 4, we mapped the number of protein complex structures, quaternary families, and quaternary folds that were deposited in the PDB in last 20 years. There has been a steady increase in the number of solved complex structures, especially in the last 10 years since the launch of structure genomics projects [43], [44]. The increase of new quaternary folds was, however, much less pronounced. A peak of new structure folds was observed in the year of 2009 which was the last year of PSI Phase II for high-throughput structure determination, while Phase III of the project (PSI:Biology) converts the focus onto the application for biological and biomedical problems [43]. It can be expected that the fraction of new quaternary folds will keep decreasing with more protein complex structures solved. If we were to assume that in the following years the growth curve would be a mirror image of the curve in the last 20 years (with the technological advancements being offset by more of the fold space being covered up), it will take roughly 25 years from now to reach approximately 4,000 unique quaternary folds or a complete set of possible quaternary folds in nature.

## Discussion

Protein quaternary structure universe dictates possible ways that proteins interact with each other. Despite the extensive analysis on the universe of protein tertiary folds for which a general consensus exists, very few such studies were conducted on protein-protein complexes. Using a non-redundant set of protein dimeric structures in the PDB and the complex structural alignment tool MM-align, this study proposed a quantitative estimate of quaternary folds possibly existing in nature.

First, a new scoring function, rTM-score, was introduced to measure the “similarity” between complex structures, which accounts for both chain orientation and monomer structural similarity into a single sensitive parameter. All non-redundant complex structures in the PDB screened at a sequence identity cutoff 90% were classified into “quaternary families” by mapping both sequences of each dimer onto the Pfam database. The 3,629 unique quaternary families were thereby clustered by rTM-score into 1,761 quaternary folds, with the largest cluster comprising 47 complex structures. About 60% of the structures were found to be structural orphans, indicating that the protein complex structure library is largely incomplete. A power-law dependence was observed between the cluster size and the number of clusters, which may implicate a cascade mechanism in the evolution of the protein-protein complexes [34], [35].

Based on the maximum probability principle, the number of possible quaternary structure folds in nature was estimated. The number of folds in our estimation varies when the number of quaternary families changes which follows a strict logarithmic dependence. If we assume that the number of quaternary families in nature is similar to the number of monomeric protein families, it was estimated that the number of expected quaternary folds in nature is approximately 4,000–5,000. This number is about two times lower than the previous estimation [17] that defined protein folds based on a sequence identity cutoff. Based on the definition of rTM-score >0.5, the rate of quaternary fold determination is low, i.e. 130 per year in last 6 years. This means we need about a quarter of century before a complete set of quaternary protein structures can be experimentally solved under the current fold solution rate.

There are several uncertainties in our model which can be improved in the future studies. First, quaternary structural folds are defined by rTM-score >0.5, which only accounts for global topology and corresponds to the complexes of the same chain-orientation and similar monomer fold (TM-score >0.5). A statistical study similar to TM-score [25] is needed to establish a more quantitative relation of rTM-score cutoffs and other measurements including interface contacts [45]. Second, the current estimation was built on the assumption that the structures in the PDB are a random subset of complexes in nature. This assumption may not strictly hold true because the observed PDB complex structures are often biased due to the difficulties in experimental determination and the research interest of the structural biology community. Third, our analysis focused mainly on physically stable complexes. This literally means that while non-obligate transient protein-protein interactions play an important role in signal transduction, electron cascades and other essential physiological processes [46], they were not considered for this study unless they were stable enough to be co-crystallized and present in the PDB. Moreover, although we have applied several filters to exclude crystallization artifacts, there are still a considerable portion of complexes that may not be bona fide dimers according to the software calculations [20], [21], [22]. Our further analysis indicates that the existence of these complexes does not essentially change the estimation of the number of quaternary folds in our model rather than the size of some families. Fourth, the number of homologous families in nature is largely unknown and our approximation using the number of monomeric families as the quaternary ones may slightly overestimate the number of the latter, since there are monomer proteins which do not participate in protein complexes and there exist also non-homologous heterodimers; they can lead to further uncertainties in the actual number of quaternary folds in nature.

Overall, despite the possible uncertainties, based on the simplicity and robustness of the model calculations and the comprehensive analysis of structural and sequence databases, our data provide the first quantitative estimation of the number of protein-protein interaction types in nature on a structural base of global topology. Given the fact that the possible phase space of protein complexes is almost infinite, it is striking that there are only several thousand possible quaternary folds, which demonstrates that the protein-protein interactions are highly specific. For instance, millions of antibodies interact with similar amount of antigens through the similar CDR locations, with the structural complexes sharing only a few unique conformations [47]. This convergence of quaternary folding space is consistent with the finding by Gao and Skolnick who recently showed that protein interfaces converge to 1,000 distinct types [48]. The limit of quaternary folding space should be mainly due to the evolutionary pressures and functional requirements of the protein-protein interactions, as well as the physical stability of these complex structures. The results presented should have importance implication to both protein-protein structure modeling and the forthcoming structure genomics of protein-protein complexes [16]. In particular, since protein quaternary folds are limited, many protein-protein interactions must share similar scaffolds, which provides important facilities to solve the protein complex structure modeling problem by the combination of template-based structure modeling [12], [13], [14] and efficient experimental structure solutions. Since a complete coverage of quaternary folding space still needs a long period of time (∼25 years), a highly selective determination of unique protein complex structures is essential to speed up the process.

## Supporting Information

Figure S1 TM-score versus rTM-score of complex structures.
(TIF)

Figure S2 Illustrative examples to highlight difference between TM-score and rTM-score values.
(TIF)

Text S1 Correlation between TM-score and rTM-score.
(DOC)
