# Supplementary figures and images for: How Many Protein-Protein Interactions Types Exist in Nature?
Source: PLoS One. 2012 Jun 13;7(6):e38913. doi: 10.1371/journal.pone.0038913 (PMC3374795; doi:10.1371/journal.pone.0038913)

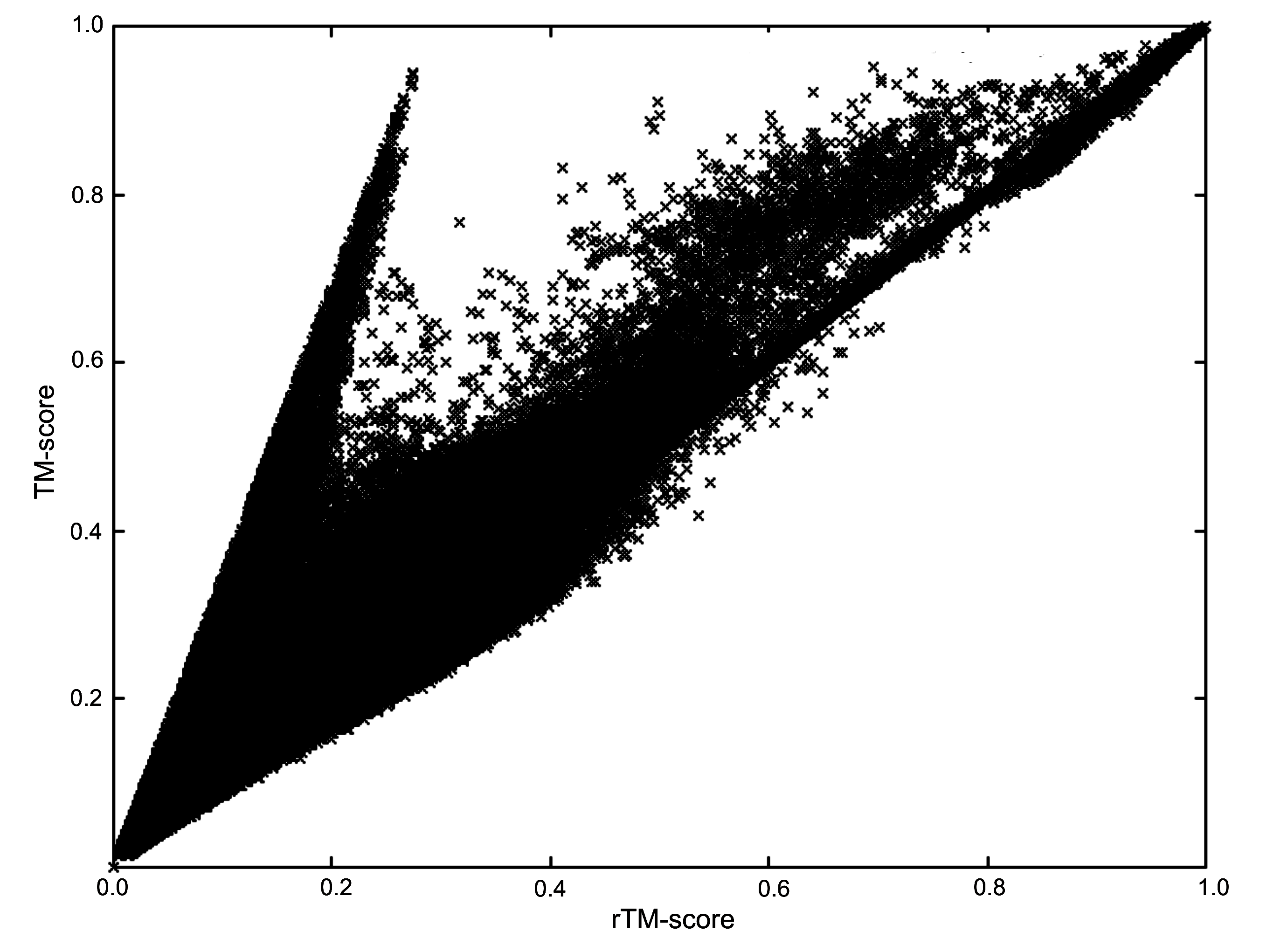

Supplement: Figure S1 — TM-score versus rTM-score of complex structures. (TIF) [file pone.0038913.s001.tif]

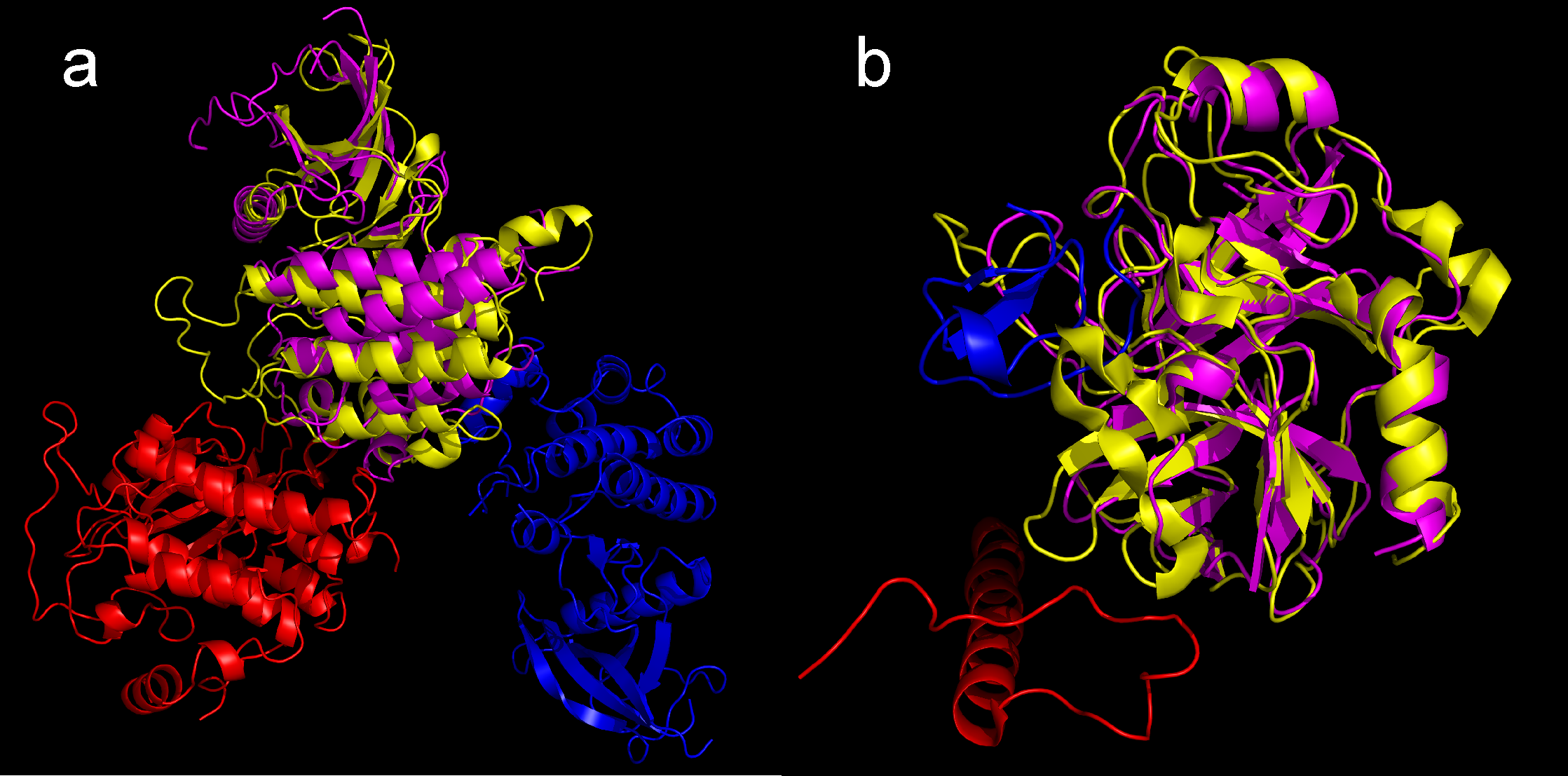

Supplement: Figure S2 — Illustrative examples to highlight difference between TM-score and rTM-score values. (TIF) [file pone.0038913.s002.tif]
