# Supplementary material for: How Many Protein-Protein Interactions Types Exist in Nature?
Source: PLoS One. 2012 Jun 13;7(6):e38913. doi: 10.1371/journal.pone.0038913 (PMC3374795; doi:10.1371/journal.pone.0038913)
Supplement: Text S1 — Correlation between TM-score and rTM-score. (DOC) [file pone.0038913.s003.doc]

**Supporting Information**

**I. Correlation between TM-score and rTM-score**

To examine the relationship of TM-score with rTM-score, a new scoring function defined to simultaneously assess the structural similarity between individual subunits of two complexes as well as their relative orientation, we screened the complete PDB library at 70% sequence identity cutoff to pick 6,306 non-redundant complexes. We then made an all-to-all comparison of 6306×6306 structure alignments by MM-align. Figure S1 shows the data of TM-score versus rTM-score as defined by Eqs. 3 and 4. As expected, the TM-score and rTM-score values are highly correlated with a Pearson correlation coefficient 0.947.

Despite the strong correlation, a significant subset of alignments exists where the value of the TM-score is inconsistent with that of rTM-score (see the points away from the diagonal line). The mismatch happens on both homodimers and heterodimers. As an illustrative example, in Figure S2a we show the structural superposition of two homodimeric complexes from the casein kinase (PDBID: 1cki) and the fibroblast growth factor receptor (PDBID: 1fgk). Individually, the monomer chains of the two dimers are structurally analogous with an average monomer TM-score =0.824; but the relative orientation of the chains are completely different. As a result, the TM-score between the two homodimer complexes are 0.442, close to the cutoff of the same fold proteins^19^. However, the rTM-score is low at only 0.216, since it is more sensitive to the chain orientations.

Figure S2b is another example of TM-score/rTM-score mismatch from two heterodimers, the thrombin-fibrinogen complex (PDBID: 2a45) and EETI-II-porcine trypsin (PDBID: 1h9h). The size of the ligand and receptor is very different in both complexes, i.e. the receptors of 2a45 and 1h9h have 258 and 220 residues and the ligands have 48 and 30 residues, respectively. The TM-score of the complexes is then dominated by the similarity of the receptor structures and have a very high value of 0.831, which does not reflect the fact that the orientation and the structure of the ligands are completely different. Accordingly, the rTM-score of the two complexes is low (=0.253).

Overall, there are 4,771,938 cases among the 6,306×6,306 alignments (~12%) where the TM-score is found to be significantly higher than the rTM-score by at least 0.25; 156,625 cases have the TM-score higher than rTM-score by at least 0.5. In these cases, TM-score does not correctly reflect the orientation differences of the complexes. The maximum difference observed was 0.669 for the complexes of PDBIDs 1omw and 1xhm. Since rTM-score is more sensitive to the global topology and chain orientation of the complexes, we use rTM-score to analyze the complex structure data in the following.

**FIGURE LEGEND:**

**Figure S1.** TM-score versus rTM-score of complex structures. Data are generated by an all-to-all comparison on 6,306 non-redundant complex structures from DOCKGROUND. The structural alignments were generated by MM-align ^15^.

**Figure S2**: Illustrative examples showing significant difference between TM-score and rTM-score values. (a) Superposition of two homodimers from Casien Kinase (PDBID: 1cki) and Fibroblast growth factor (1fgk) with TM-score = 0.442 and rTM-score = 0.216. (b) Superposition of two heterodimers from Thrombin-Fibrinogen complex (2a45) and EETI-II-porcine trypsin (1h9h) with TM-score=0.831 and rTM-score=0.253. The cartoon of the receptor and ligand is shown in red and blue in one complex, and yellow and magenta in another complex.
